# Evaluation of neodymium isotope analysis of human dental enamel as a provenance indicator using 10^13^ Ω amplifiers (TIMS)

**DOI:** 10.1016/j.scijus.2019.02.001

**Published:** 2019-05

**Authors:** E. Plomp, I.C.C. von Holstein, J.M. Koornneef, R.J. Smeets, J.A. Baart, T. Forouzanfar, G.R. Davies

**Affiliations:** a Department of Earth Sciences, Vrije Universiteit Amsterdam, de Boelelaan 1085, 1081 HV, Amsterdam, the Netherlands; b Department of Oral and Maxillofacial Surgery/Oral Pathology, VU University Medical Center (VUMC), De Boelelaan 1117, 1081 HV, Amsterdam, the Netherlands; c Academic Centre for Dentistry Amsterdam (ACTA), Gustav Mahlerlaan 3004, 1081 LA, Amsterdam, the Netherlands

**Keywords:** Neodymium isotopes, Strontium isotopes, Human, Provenance, Enamel

## Abstract

Human provenance studies employing isotopic analysis have become an essential tool in forensic and archaeological sciences, with multi-isotope approaches providing more specific location estimates compared to single isotope studies. This study reports on the human provenancing capability of neodymium isotopes (^143^Nd/^144^Nd), a relatively conservative tracer in the environment. Neodymium isotope ratios have only recently been determined on human remains due to low concentrations in human dental enamel (ppb range), requiring thermal ionisation mass spectrometry (TIMS) using 10^13^ Ω resistors. Dental elements (third molars) from 20 individuals born and raised in the Netherlands were analysed for Nd concentration (*n* = 12) and Nd isotope ratios (*n* = 15). The geological control on Nd isotope composition was examined using coupled Nd-Sr isotope analysis of the same third molar. Teeth from different geological environments were also analysed (Caribbean, Columbian, and Icelandic, *n* = 5). Neodymium elemental concentrations in dental elements ranged between 0.1 and 7.9 ppb (median 0.5 ppb). The Dutch ^143^Nd/^144^Nd ratios of the provinces of Limburg and Friesland were between 0.5118 and 0.5121, with Dutch ^87^Sr/^86^Sr ratios in agreement with the previously established local range (0.708–0.710). The current findings were compared to previously published results on Nd concentration and composition from Dutch individuals. The concentration of Nd and ^143^Nd/^144^Nd ratios were weakly correlated (R^2^ = 0.47, *n* = 17) in Dutch human dental enamel. The majority (*n* = 25, 83.3%) of individuals had Nd and Sr isotope values isotopically indistinguishable from the geological environment in which their third molars formed and mineralised. However, the Nd isotope ratios of the Icelandic individual and several Dutch individuals (*n* = 4) suggested that Nd in enamel is not solely influenced by geological environment. In order for neodymium isotopes to be quantitatively applied in forensic and archaeological settings further analyses of individuals from various geographical regions with well-defined dietary Nd isotope data are required.

## Highlights

• First evaluation of the potential of neodymium isotopes for human provenancing
• Provides insight in Nd concentration and isotope ratios in human dental enamel
• First study presenting Nd isotope ratios from multiple geological settings
• First study combining ^143^Nd/^144^Nd and ^87^Sr/^86^Sr ratios of the same dental element
• The use of 10^13^ Ω resistors allows for greater precision analysis of small samples

## Graphical abstract

**Figure:**
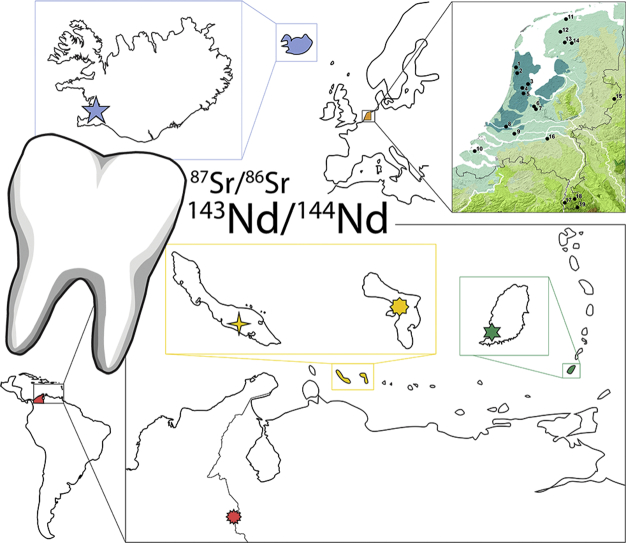

## 1 Introduction

Isotope analysis of human tissues, such as hair, nails, skeletal and dental elements, can give insight into the mobility profile of an individual, as these tissues reflect the isotopic values of the environment in which the individual lived at the time of tissue formation. This mobility profile does not permit direct identification of unidentified victims, but provides forensic intelligence to construct a profile that may lead to identification of an individual when coupled with osteological and forensic information [1]. Isotopic systems which preserve environmental information in human tissue include strontium (^87^Sr/^86^Sr), oxygen (δ^18^O), hydrogen (δ^2^H) and lead (^204^Pb, ^206^Pb, ^207^Pb, ^208^Pb). These isotopic systems have been applied to obtain information on the geographical region of origin and/or recent movements of individuals in forensic [[2], [3], [4], [5], [6], [7], [8], [9], [10]] and archaeological contexts [[11], [12], [13], [14], [15], [16], [17], [18]]. The integration of multiple isotopic systems has been shown to be a powerful approach [[2], [3], [4],^12^,^16^,^17^].

This study examines the potential use of an additional isotope system for human provenancing: neodymium (Nd) isotope ratios. The addition of another isotopic system would allow for increased geographical discrimination as it provides complimentary information to the other isotopic systems. The potential for Nd analysis to be used for human provenancing is examined by evaluating the geological control on ^143^Nd/^144^Nd ratios in human enamel. Teeth (third molars) were sampled from individuals that resided in geological contexts with various isotopic compositions (the Netherlands, the Caribbean, Columbia and Iceland). Neodymium elemental concentrations as well as neodymium and strontium isotope ratios in modern human dental enamel were analysed using thermal ionisation mass spectrometry (TIMS) employing 10^11^ Ω and 10^13^ Ω amplifiers. The 10^13^ Ω amplifiers are essential for the measurement of small Nd samples, allowing reliable analysis of samples as small as 100 pg. These small Nd samples cannot be successfully analysed with the default 10^11^ Ω amplifiers that require >1 ng Nd for analysis [19,20]. The findings of this study were examined and compared to previously published results on Nd and composition from Dutch individuals [19] to evaluate the application of Nd isotopes for human provenancing.

## 2 Isotope method background

Despite widespread use, the application of isotopic analysis for human provenancing has several limitations:

(1) Sample selection may be biased by availability of human tissues recovered from forensic cases and archaeological sites. Moreover, tissues retrieved from these contexts can be affected by diagenesis, with the exception of dental enamel [21].
(2) Accurate evaluation of isotopic data from human tissues requires bioavailable background data such as isoscapes: maps reporting bioavailable isotopic ratios in an environment. Unfortunately, detailed isoscapes are rarely available as they require extensive sampling and sophisticated modelling approaches [12,[22], [23], [24], [25], [26], [27]].
(3) The import of food grown in geological environments different to the residential area of an individual may influence the isotopic values of an individual, particularly in globalised modern societies [28,29].
(4) Anthropogenic Pb [30] or marine Sr [31] can overwrite the geological signature found in human remains.
(5) Identical isotopic signatures can be obtained from individuals originating from different geographic areas due to similar (geological) environments resulting in poor provenance discrimination.

These last three limitations could be partially addressed by applying a multi-isotope approach as the various isotopic systems reflect different parts of the environment: geology (Sr, Pb), drinking water (O, H) and pollution (Pb). Taken together, multiple isotope systems could provide greater spatial resolution by distinguishing between environments where a single isotopic system cannot [2,12,16]. This study examines the potential of neodymium isotope ratios (^143^Nd/^144^Nd) for human provenancing. The variation of ^143^Nd/^144^Nd and ^87^Sr/^86^Sr ratios in human enamel of the same dental element are determined to understand the geological control on the isotopic values.

Strontium isotope analysis (^87^Sr/^86^Sr) of human tissues is an established provenancing tool for modern and archaeological individuals [2,4,13,14,16,32,33]. Strontium enters the human body through the diet, as vegetation and bodies of water take up strontium predominantly derived from the local geology [32]. Strontium ratios vary spatially dependent on rock type and age of formation. Low ^87^Sr/^86^Sr ratios (<0.704) are generally found in geological young deposits (<1–10 Mya) and high ^87^Sr/^86^Sr ratios (>0.710) are found in older rocks (>100 Mya) [32]. Strontium ratios in human tissue can therefore be an indication of provenance when locally grown food dominates the diet.

Neodymium isotope ratios (^143^Nd/^144^Nd) have been successfully applied to provenance glass archaeological artefacts [[34], [35], [36]] and modern animal bones [37]. Neodymium is a light rare earth element (LREE) which varies geologically in ^143^Nd/^144^Nd ratios as a result of the rock age, rock type/composition, and tectonic settings [38,39]. Generally, older geological depositions have lower ^143^Nd/^144^Nd ratios compared to recently formed deposits, with ^143^Nd/^144^Nd ratios typically ranging between 0.510 and 0.514 [40]. Neodymium isotope ratios are transferred from rocks to the vegetation and bodies of water, entering the human body through diet, inhalation and potentially dermal contact [19,41,42]. It is expected that Nd isotopes are not isotopically fractionated during their uptake by the human body, thus reflecting the environmental ratios of the food, water and dust consumed [37,39]. Due to the low concentrations of Nd present in human tissues (<0.7 ppm) there have been few studies addressing neodymium in biological systems (see Plomp et al. [19] for a summary). Low Nd concentrations (0.1 to 58.0 ppb [19,43]) in dental enamel limit its analysis and application to human provenancing. The low Nd concentrations in human tissues can be explained by (1) the low levels of bioavailable Nd in the food chain (ppb range), as vegetation and bodies of water take up limited Nd from the underlying geology [[44], [45], [46]], (2) the moderately toxic nature of Nd [47,48] and lack of physiological or biological function of Nd in the human body [47] and (3) the trivalent ion (Nd^3+^), which is incompatible with calcium (Ca^2+^) substitution in the hydroxyapatite crystal lattice of human bone and teeth [19,47].

Neodymium is a promising candidate for human provenancing due to its different geochemical characteristics in comparison to Sr [37,49]. Nd isotope ratios from coastal locations are less susceptible than Sr to sea-spray effects (whereas geological Sr isotope ratios are dominated by seawater ^87^Sr/^86^Sr [32]) as the Nd contents in water are low (typically 10–30 pmol/L) and the residence time of Nd in the ocean is relatively short (in comparison to Sr) [50]. Hence, the ^143^Nd/^144^Nd ratios found in oceans reflect the surrounding geology [51,52]. The diminished influence of sea-spray makes Nd isotope analysis a promising candidate for human provenance analyses, particularly in coastal regions.

## 3 Material

**Fig. 1:**
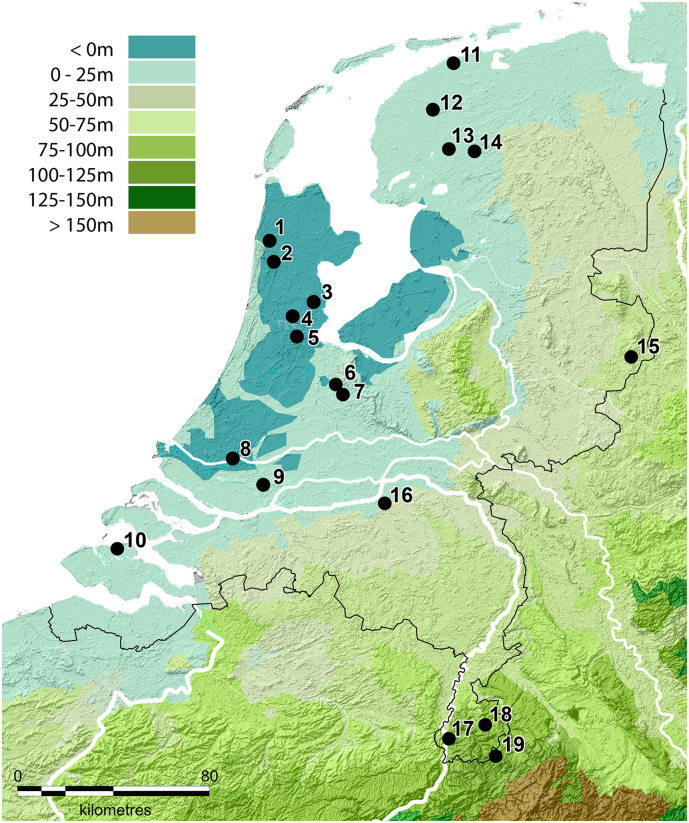
A topographic map of the Netherlands with localities where the individuals lived during formation and mineralisation of their third molars. 1 = Warmenhuizen, 2 = Alkmaar, 3 = Purmerend, 4 = Zaandam, 5 = Amsterdam, 6 = Maarsen, 7 = Utrecht, 8 = Rotterdam, 9 = Dordrecht, 10 = Kortgene, 11 = Holwerd, 12 = Leeuwarden, 13 = Oldeboorn, 14 = Lippenhuizen, 15 = Enschede, 16 = Den Bosch, 17 = Maastricht, 18 = Heerlen, 19 = Vaals.

**Fig. 2:**
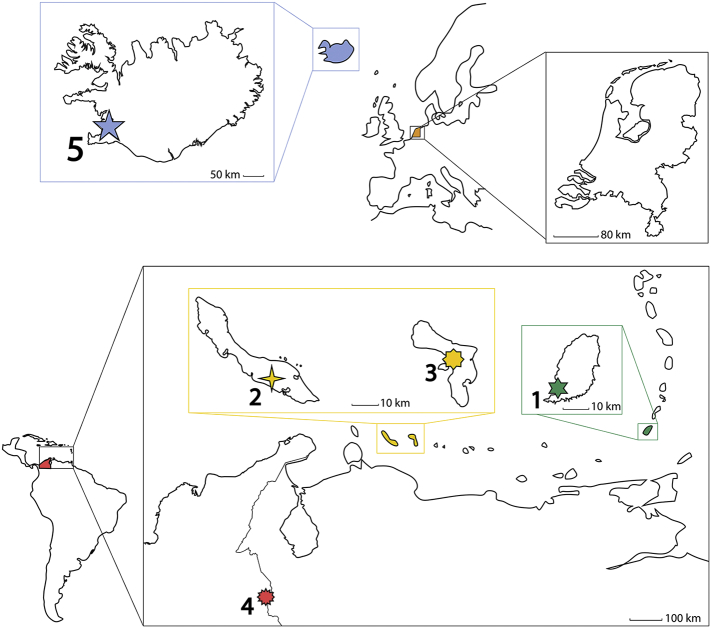
A map of Europe and the Caribbean region indicating the location where the individuals lived during mineralisation of their third molars. 1 = St. George's (Grenada), 2 = Willemstad (Curaçao), 3 = Kralendijk (Bonaire), 4 = Cúcuta (Columbia), 5 = Reykjavik (Iceland).

Neodymium and strontium isotope analysis was performed on third molars (M3) from individuals who were known to have been residentially stable during the period of third molar formation and mineralisation. The enamel of third molars is formed between the age of 8–16 years [53] and isotopic results are therefore representative of the environment in which the individual lived during that time period. Analysis was performed on the mineralised outer surface of the teeth, the enamel. Third molars were donated by inhabitants of the Netherlands (*n* = 56, including data from Plomp et al. [19]) (Fig. 1), the Caribbean (*n* = 3), Columbia (*n* = 1) and Iceland (n = 1) (Fig. 2) of which the geological environments were expected to produce a sufficient range of Nd and Sr isotope compositions (see Section 4: Background). The Dutch residents were grouped based on geographical residence and geological substrate at the age of 8–16 years. Dutch individuals lived in the provinces of North Holland (primarily Amsterdam) (*n* = 22), South Holland (primarily Rotterdam) (*n* = 13), Friesland (*n* = 7), Limburg (*n* = 9), and other regions in the Netherlands (*n* = 5) (Fig. 1). The teeth from North Holland/Amsterdam were previously analysed for Sr, O, Pb [2] and Nd isotopes [19]. The Nd results for the South Holland/Rotterdam individuals were previously published in Plomp et al. [19]. The current study reports the Sr and Nd results for the Friesland and Limburg provinces and the Nd results for other regions in the Netherlands (*n* = 20), as well as the Sr results for South Holland.

The analyses of the teeth were approved by the Medical Ethics Review Committee of the VU University Medical Center. Questionnaires provided anonymised background information on the donors and information on the geographic location of the individual at the time of tooth formation and mineralisation, as well as diet, health, smoking and exercise habits. Precise background information (other than geographic location during enamel mineralisation) was not available for the Icelandic and Grenadian individuals due to collection of these teeth outside the Netherlands (collection which was not covered by the Medical Ethics application).

## 4 Background

The Netherlands is a European country (Fig. 1, Fig. 2) formed of geology consisting of Holocene deposits in the northwest, and Pleistocene areas in the south [54]. The surface deposits comprise marine, fluvial (Rhine, Meuse and Scheldt rivers) and glacial sediments of Quaternary age (2.6 Ma-present), with local loess and peat layers [55,56]. Limited neodymium background sampling exists for the region. Rhine River sediment data range from ^143^Nd/^144^Nd = 0.51198 to 0.51217 (*n* = 18 [57,58]) and is used here as a general indication for the local Dutch neodymium isotope variation. Neodymium isotope ratios in Dutch human enamel ranges from ^143^Nd/^144^Nd = 0.51187–0.51259 (*n* = 20 [19]). The Sr local range (^87^Sr/^86^Sr = 0.708–0.710 [2]) is based on studies reporting human scalp hair and enamel data from modern Dutch individuals, tap water and soil/street dust. Dutch archaeological background samples exhibit higher variation with ^87^Sr/^86^Sr ratios up to 0.711 [14].

The Caribbean samples originate from two distinct geological environments. Grenada is the southernmost island of the Lesser Antilles Island Arc (Fig. 2) where the subduction induced mantle-derived magmas are contaminated with sediment at depth and in the crust [59]. These sediments are derived from the Orinoco and Amazon deltaic systems [59]. Volcanism on Grenada has ^143^Nd/^144^Nd and ^87^Sr/^86^Sr ratios in the range 0.5123–0.5126 and 0.7038–0.7064 respectively [60]. The island is subjected to sea-spray influence (^87^Sr/^86^Sr = 0.7092 [32]) and a significant dust flux from North Africa characterised by ^143^Nd/^144^Nd and ^87^Sr/^86^Sr ratios between 0.5116 and 0.5126 and 0.715–0.718 respectively [[61], [62], [63], [64]]. Bonaire and Curaçao are part of the Cretaceous Caribbean Flood basalt province, but both islands include more recent carbonate sediments. Estimated bioavailable Sr isotope ratios range from 0.703–0.709 [12] and estimated ^143^Nd/^144^Nd varies from 0.5120 in the limestone regions to values as high as 0.5130 in the volcanic regions [52,65]. Like Grenada, the islands of Bonaire and Curaçao are influenced by sea-spray and North African dust aerosol deposition [[61], [62], [63]].

The individual from Colombia lived in Cúcuta, a city at the Venezuelan border in the Maraicaibo Basin lying outside the active volcanic zones of South America (Fig. 2). The region is made up of Quaternary and Tertiary sediments derived from Cretaceous to Precambrian basement. Modelling estimates the local bioavailable Sr > 0.710 [25]. The basement in the central and eastern Cordillera was metamorphosed at ~1.0 Ga and the limited data indicates this region has depleted mantle Nd model ages (T_DM_) between 1.5 and 2.0 Ga and hence a ^143^Nd/^144^Nd of ~0.5120 [66].

The individual from Iceland lived in the Reykjavik region (Fig. 2) where the local basement lavas have ^143^Nd/^144^Nd and ^87^Sr/^86^Sr ratios in the range 0.5130 to 0.5131 and 0.7031 to 0.7032 respectively [67,68]. Iceland receives major sea-spray influence [69,70] but dust flux is limited.

## 5 Methods

### 5.1 Sample preparation

The enamel was sampled, chemically processed and analysed at the Faculty of Science, Vrije Universiteit Amsterdam. Sample preparation and procedures are described in detail in Plomp et al. [19]. The enamel was sampled using a dental micro-drill fitted with a cleaned diamond-tipped rotary burr and blade (Minilor Perceuse). Sample weight for Nd composition in this study ranged from 222 to 1464 mg (average = 733 mg, *n* = 20) of which 1–2% aliquots were taken for Sr analysis for the individuals from South Holland, Limburg, Friesland, the Caribbean, Columbia and Iceland (*n* = 25). If two third molars were available from a single donor, the enamel from both teeth was combined to increase available sample size.

### 5.2 Chemical separation

Sample dissolution and chromatographic separation was performed in a class 100 clean laboratory. All PFA laboratory equipment was cleaned according to standard procedure [19]. In order to assess the variability introduced by the laboratory procedures a synthetic tooth standard (TSTD) was used [19]. TSTD aliquots were processed on 0.75 and 1.3 mL TRU-resin columns (10 mL, 4 ng Nd, 1000 mg CaHPO_4_) and Sr columns (0.05 mL, 500 ng Sr, 5 mg CaHPO_4_).

In order to determine the range of Nd concentrations in human teeth, isotope dilution was performed on a subset of the samples before dissolution [[71], [72], [73]]. This method allows for the simultaneous measurement of both Nd elemental concentration and Nd isotope composition of a single sample. The enamel was dissolved in two steps using 6.5 N HCl and a mixture of 6.5 N HCl and 14.0 N HNO_3_ before being taken up in 10 mL 2.0 N HNO_3_ for column extraction [19]. Neodymium extraction followed the procedure described in Plomp et al. [19] (available on protocols.io: dx.doi.org/10.17504/protocols.io.xzmfp46), using TRU-resin columns with resin volumes ranging from 0.75 mL (samples <550 mg) to 1.3 mL (samples >550 mg). After LREE extraction, Nd was separated from the other LREE using Ln-resin (Eichrom Technologies) following standard procedure [71].

An aliquot of 100–200 μL (depending on sample size) was taken from the samples for Sr analysis, which was separated using pipette tips (with 30 μm pore size frit material [74]) and 100 μL Sr-spec resin.

### 5.3 TIMS

Neodymium and Sr analyses were performed on a Thermo Scientific Triton *Plus* TIMS [20]. Standards and samples were loaded on out-gassed Re filaments in 1–2 μL 10% HNO_3_ with 1 μL H_3_PO_4_ for Nd (see Koornneef et al. [75] for details) and 50% of the Sr fraction in 1 μL 10% HNO_3_, with 1.5 μL TaCl_5_ for Sr.

Neodymium analyses were performed using 10^13^ Ω resistors fitted to the amplifier system (see Koornneef et al. [20] for details) and 10^11^ Ω resistors if enough sample was available, following procedures described in detail in Plomp et al. [19]. ^143^Nd/^144^Nd ratios were corrected for mass-fractionation to ^146^Nd/^144^Nd = 0.7219. A minimum of 70 scans were collected for each analysis on 10^13^ Ω amplifiers, and 60 scans for 10^11^ Ω amplifiers. Larger standards and samples (>1 ng) were stopped after 300–400 cycles using 10^13^ Ω resistors and re-analysed using 10^11^ Ω amplifiers to check for precision and accuracy. Repeat sample analyses were within analytical error [19].

Standards were measured to check for accuracy and reproducibility. Small aliquots, 100 pg, of an internal standard, CIGO (see Koornneef et al. [75] for more details on CIGO), measured with 10^13^ Ω amplifiers (^143^Nd/^144^Nd = 0.511344 ± 70 2SD, *n* = 40) were in agreement with 250 ng CIGO measurements determined using the 10^11^ Ω amplifiers (^143^Nd/^144^Nd = 0.511328 ± 9 2SD, *n* = 50). JNdi-1 results using 10^11^ Ω amplifiers gave an average ^143^Nd/^144^Nd of 0.512096 (±61 2SD, *n* = 22). Analysis of 0.5–4.0 ng Nd TSTD using 10^13^ Ω amplifiers (^143^Nd/^144^Nd = 0.512134 ± 72 2SD, *n* = 81) are in agreement with measurements using 10^11^ Ω amplifiers (^143^Nd/^144^Nd = 0.512125 ± 61 2SD, *n* = 49). Total procedural blanks yielded 1.1 ± 1.7 pg for Nd (*n* = 56). Previous research in our lab demonstrates that blank contributions to samples as small as 30 pg Nd are negligible [71,72], hence blank corrections were not required.

Strontium analyses were performed using default 10^11^ Ω resistors. Isotope ratios were corrected for mass-fractionation to ^86^Sr/^88^Sr = 0.1194. Standards measured for the period of a year resulted in ^87^Sr/^86^Sr = 0.710247 ± 17 (2SD, *n* = 51, 100–200 ng) for NBS987 (accepted ^87^Sr/^86^Sr = 0.710248) and ^87^Sr/^86^Sr = 0.707854 ± 19 (2SD, *n* = 97) for the internal TSTD (first publication of ^87^Sr/^86^Sr ratios of the inhouse TSTD). The procedural blanks yielded an average of 24.7 pg strontium (±38.9, *n* = 26), a negligible amount compared to the average amount of strontium present in enamel samples (100–800 ng [32]).

## 6 Results

### 6.1 Neodymium elemental concentration

**Fig. 3:**
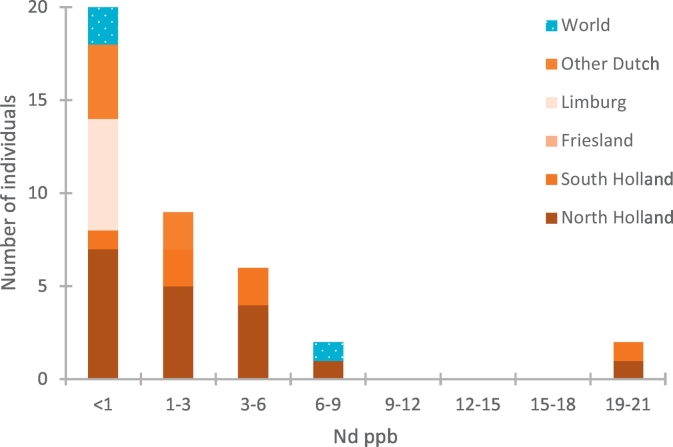
Neodymium concentrations of individuals from North Holland (*n* = 18), South Holland (*n* = 6), Limburg (n = 6), other parts of the Netherlands (n = 6), and other parts of the world (n = 3) (total n = 39). Concentration results from the individuals from North and South Holland (*n* = 23) were previously published by Plomp et al. [19].

**Fig. 4:**
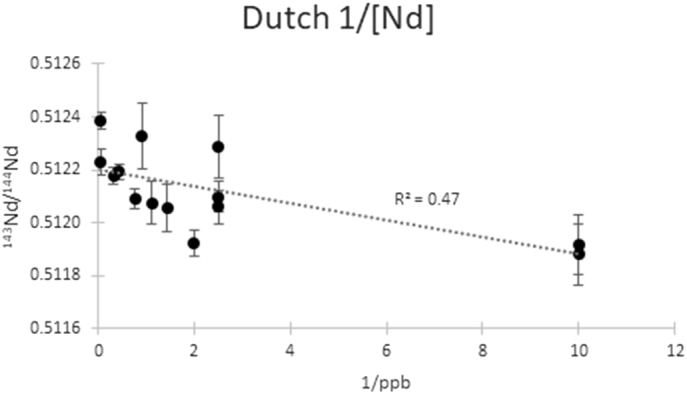
^143^Nd /^144^Nd vs the reciprocal of neodymium content (ppb) for Dutch samples (1/[Nd], n = 17). The two samples from Limburg containing 0.1 ppb Nd are at the right extreme of the graph.

This paper reports new Nd concentration data from the enamel of a subset of the individuals (*n* = 15; 12 Dutch individuals and 3 individuals from Grenada, Bonaire and Colombia) (Table 1). Concentrations for these 15 individuals ranged from 0.1 ppb to 7.9 ppb, with a median of 0.5 ppb. These data are considered together with Nd concentration data from Dutch individuals previously published (total *n* = 39, Plomp et al. [19], Fig. 3). For the majority of the individuals tested, enamel contains <1 ppb Nd (*n* = 20, 51.3%, Fig. 3). The total Dutch sample set records a weak correlation between Nd concentration and isotopic composition (R^2^ = 0.47, *n* = 17, Fig. 4).

**Table 1:** Nd concentration and isotope composition of third molars (*n* = 47). Nd was analysed using 10^13^ resistors and Sr isotope composition using 10^11^ resistors.

Group	Sample	Location	Sample size (mg)	Nd content (ppb)	^143^Nd/^144^Nd ± 2SD	εNd	^87^Sr/^86^Sr ± 2SD
North Holland	1A-22	Amsterdam	530		0.512044 ± 44^b^	−11.6^b^	
	2A-20	Warmenhuizen	511	1.3^b^	0.512091 ± 37^b^	−10.7^b^	0.709267 ± 7^a^
	3A-24	Amsterdam	529	2.3^b^	0.512193 ± 29^b^	−8.7^b^	0.709378 ± 7^a^
	4A-H	Purmerend	320	19.8^b^	0.512229 ± 47^b^	−8.0^b^	
	5A-25	Amsterdam	350	0.7^b^	0.512056 ± 91^b^	−11.4^b^	
	6A-10	Amsterdam	276	3.1^b^	0.512175 ± 32^b^	−9.0^b^	0.709315 ± 6^a^
	7A-13	Amsterdam	279	0.4^b^	0.512098 ± 59^b^	−10.5^b^	0.709409 ± 9^a^
	8A-27	Alkmaar	273		0.512185 ± 188^b^	−8.8^b^	0.709367 ± 7^a^
	9A-28	Amsterdam	513		0.512380 ± 119^b^	−5.0^b^	
	10A-18	Amsterdam	418	0.4^b^	0.512288 ± 119^b^	−6.8^b^	0.709584 ± 6^a^
	11A-15	Amsterdam	431		0.512589 ± 80^b^	−1.0^b^	0.709441 ± 4^a^
	12A-9	Amsterdam	299	1.1^b^	0.512330 ± 124^b^	−6.0^b^	0.709231 ± 4^a^
South Holland	28R-14a	Dordrecht	429	21.0^b^	0.512388 ± 32^b^	−4.9^b^	0.709153 ± 6
	29R-11	Rotterdam	1233⁎		0.512080 ± 29^b^	−10.9^b^	0.709375 ± 11
	30R-13	Rotterdam	870⁎		0.511869 ± 28^b^	−15.0^b^	0.709409 ± 9
	31R-2	Rotterdam	482		0.512048 ± 28^b^	−11.5^b^	
	32R-3	Rotterdam	746⁎		0.511945 ± 48^b^	−13.5^b^	
	33R-9	Rotterdam	589		0.511972 ± 100^b^	−13.0^b^	
	34R-5	Dordrecht	582		0.511987 ± 105^b^	−12.7^b^	0.709061 ± 8
	35R-9	Rotterdam	636		0.512020 ± 166^b^	−12.1^b^	0.709821 ± 9
Friesland	36-F1	Lippenhuizen	1310⁎		0.511959 ± 30	−13.2	0.709432 ± 9
	37-F3	Holwerd	652		0.511938 ± 130	−13.7	0.709619 ± 9
	38-F4	Leeuwarden	452		0.512011 ± 94	−12.2	0.708934 ± 10
	39-F8	Leeuwarden	510		0.511820 ± 107	−16.0	0.709469 ± 7
	40-F11	Leeuwarden	570		0.512046 ± 63	−11.5	0.709230 ± 9
	41-F12	Oldeboorn	361		0.512048 ± 35	−11.5	0.709122 ± 9
	42-F13	Leeuwarden	415		0.511928 ± 60	−13.8	0.709337 ± 9
Limburg	43-R6	Maastricht	1464⁎		0.511999 ± 29	−12.5	0.708942 ± 7
	44-M4	Maastricht	384		0.511820 ± 49	−16.0	0.709596 ± 7
	45-M5	Maastricht	712		0.511973 ± 56	−13.0	0.709644 ± 10
	46-M10	Maastricht	66	0.1			
	47-M14	Maastricht	1190⁎	0.1	0.511880 ± 117	−14.8	0.709546 ± 10
	48-ZH1	Heerlen	864⁎	0.1	0.511916 ± 112	−14.1	0.709424 ± 8
	49-ZH3	Heerlen	1226⁎	0.4	0.512061 ± 64	−11.3	0.709319 ± 9
	50-ZH4	Heerlen	969⁎	0.9	0.512075 ± 80	−11.0	0.709169 ± 9
	51-ZH9	Vaals	222	0.5	0.511924 ± 50	−13.9	0.709862 ± 8
Other Dutch	52-7	Utrecht	53	0.5			0.709382 ± 6^a^
	53-3	Maarssen	59	1.8			0.708951 ± 6^a^
	54-S3a	Kortgene	56	0.8			
	55-S2b	Kortgene	33	0.8			
	56-1	Den Bosch	63	1.2			0.709611 ± 9^a^
	57-16	Enschede	41	0.5			0.709674 ± 8^a^
World	W1-Gr	Grenada (St. George's)	421	7.9	0.512773 ± 30	2.6	0.707841 ± 9
	W2-R8	Curaçao (Willemstad)	489		0.512131 ± 43	−9.9	0.709375 ± 10
	W3-B4	Bonaire (Kralendijk)	873⁎	0.6	0.512127 ± 38	−10.0	0.709256 ± 9
	W4-B16	Columbia (Cúcuta)	1036⁎	0.2	0.512043 ± 142	−11.6	0.711749 ± 9
	W5-I	Iceland (Reykjavik)	548		0.511889 ± 29	−14.6	0.708740 ± 9

εNd = [((^143^Nd/^144^Nd)sample/(^143^Nd/^144^Nd)CHUR) − 1] × 10^4^, with the accepted value of the Chondritic Uniform Reservoir (CHUR) = 0.512638 [76]. Data available in CSV format at 4TU. Centre for Research Data (http://doi.org/10.4121/uuid:d541a402-2701-47b2-ac6a-eaaa14c8c111).

⁎ 2 teeth combined.

a Results published in Font et al. [2].

b Results published in Plomp et al. [19].

### 6.2 Neodymium and strontium isotope composition

**Fig. 5:**
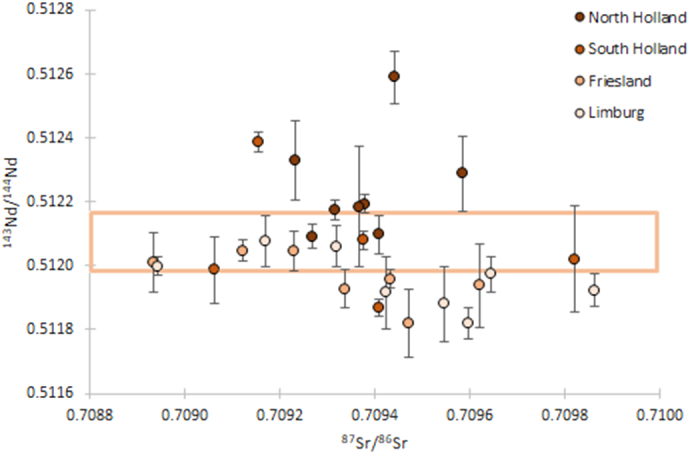
Nd and Sr isotope compositions of third molars (*n* = 28) from Dutch individuals. The Dutch isotope range is represented by the orange box based on river sediment data (Nd [57,58]), human scalp hair and enamel data from modern Dutch individuals, tap water and soil/street dust (Sr [2]). Neodymium results for the individuals from North and South Holland were previously published by Plomp et al. [19]. Strontium results for the individuals from North Holland were previously published by Font et al. [2].

**Fig. 6:**
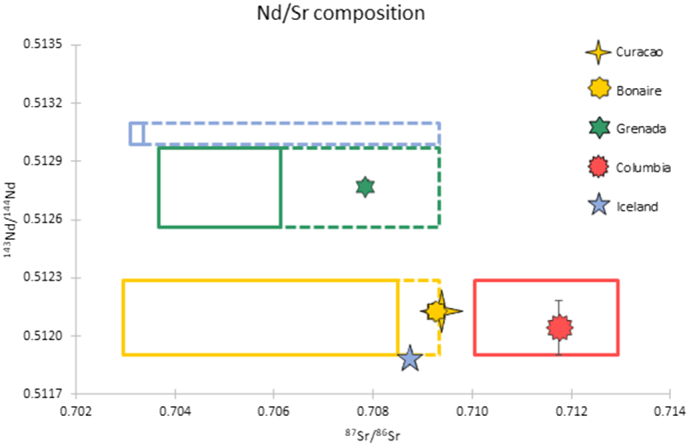
Nd and Sr isotope compositions of third molars (*n* = 5) from Caribbean, Columbian and Icelandic individuals. The expected local isotope ranges, as described in Section 4, are represented by the solid line boxes, with stippled lines representing potential Sr sea-spray influence. The standard deviations are smaller than the symbols (unless visible).

This study adds enamel Nd isotope ratios of 15 Dutch inhabitants and 5 inhabitants from Curaçao, Bonaire, Grenada, Colombia and Iceland to the existing dataset of 20 Dutch individuals [19] (Table 1). Eight samples analysed for Nd isotope ratios did not contain enough Nd for reliable analysis (<70 scans). Paired Nd and Sr isotope ratios were determined for 33 out of 40 individuals (Table 1); 28 Dutch individuals (Fig. 5) and 5 individuals from the Caribbean, Columbia and Iceland (Fig. 6). This study reports Nd and Sr results on the same fraction for 25 individuals (South Holland, Limburg, Friesland, Caribbean, Columbia and Iceland). Neodymium and strontium isotope ratios for North Holland were performed on separate sample fractions (*n* = 8 [2,19]).

The ^143^Nd/^144^Nd isotope ratios from the Dutch individuals from Limburg (n = 8) ranged from 0.51182–0.51208 (median = 0.51195) and for Friesland (*n* = 7) between 0.51182 and 0.51205 (median = 0.51196). These results are compatible with or lower than the ^143^Nd/^144^Nd isotope range found in Dutch river sediments (^143^Nd/^144^Nd = 0.51198–0.51217, *n* = 18 [57,58]) and overlap with the results of the individuals from South Holland (^143^Nd/^144^Nd = 0.51187–0.51239, n = 8) (Fig. 5). The range in ^87^Sr/^86^Sr ratios in the Dutch inhabitants (0.70894–0.70982, Fig. 5) is compatible with the defined modern Dutch range (^87^Sr/^86^Sr = 0.708–0.710 [2]).

The combined Sr-Nd isotope results from the individuals from Grenada, Curaçao, Bonaire, and Colombia are plotted in Fig. 6. Both the Nd and Sr ratios are consistent with local geology for the individual from Colombia. The individual from Grenada exhibits Nd ratios compatible with local geology, but shows an elevated Sr ratio compared to the local volcanic geology. Elevated Sr isotope ratios are also seen in the Curaçao (0.7094) and Bonaire (0.7093) samples, with Nd ratios isotopically indistinguishable from the local geology. The Iceland sample has a Nd ratio (^143^Nd/^144^Nd = 0.51189) significantly lower than the local volcanic rocks (^143^Nd/^144^Nd = ~0.5130). The individual's Sr ratio (^87^Sr/^86^Sr = 0.708740 ± 9) is also incompatible with the local volcanic geology.

## 7 Discussion

### 7.1 Nd elemental concentration variability

The low Nd concentration present in human tissue (<0.7 ppm) means that to date there are limited studies of Nd uptake into living tissue and bioavailable Nd, as well as the effect of anthropogenic contributions. Initial Nd concentration data reported low concentrations with significant variation in Dutch individuals (0.1 to 21.0 ppb, *n* = 23 [19]), in which higher Nd concentrations (19.8 and 21.0 ppb) were explained by either (1) local exposure by industrial Nd products or (2) variation in the uptake of Nd in the human body due to individual differences in physiological factors such as sex, age, and activity patterns (or a combination of the two factors). The current study reports similar low concentrations ranging from 0.1 to 7.9 ppb (*n* = 16). The 0.9 ppb median (*n* = 39) is lower than previously reported for the individuals from North and South Holland (1.2 ppb median, <1–21 ppb), as individuals from Limburg show consistently lower Nd concentration in their enamel (<1 ppb). The weak correlation between Nd elemental concentration and isotope composition (R^2^ = 0.47, Fig. 4) suggests that local geology is not the only factor controlling neodymium in dental enamel. This correlation is still evident after removal of outliers with especially low Nd concentrations (0.1 ppb, *n* = 2, R^2^ = 0.21) or elevated Nd isotope ratios (^143^Nd/^144^Nd ≥ 0.5122, *n* = 4, R^2^ = 0.64). The low Nd concentrations found in dental enamel (<9 ppb) suggest that systemic pollution is unlikely to be a factor influencing the Dutch population, as Nd concentrations are much higher in lung (>50 ppb) and hair (>160 ppb) tissues reported for individuals affected by acute Nd pollution [19,41,42]. Local Nd pollution caused by fossil fuel, fertilisers or waste combustion and metallurgic processes such as the production of magnets [44], or contact with these (electro)magnets in smart phones, computers and other electronic equipment could influence the Nd found in human tissues from specific individuals. Local anthropogenic Nd exposure cannot be excluded based on the available information of this dataset, as the originally low Nd concentrations in human enamel (<1–9 ppb) would be easily affected by such influences. Smoking does not seem to increase Nd concentration in enamel, as two reported smokers during enamel mineralisation (7A-13 and 48-ZH1) had low Nd concentrations (0.1 and 0.4 ppb respectively).

### 7.2 Geological control of Nd-Sr isotopes

To determine whether Nd isotope ratios can be used as a provenance indicator, this study examined if the local geology controlled the Nd elemental concentration and isotope composition in modern human enamel. Both ^143^Nd/^144^Nd and ^87^Sr/^86^Sr ratios of the same dental element were assessed to provide an additional geographical proxy next to information obtained from the questionnaires. The differences between an individual Nd or Sr isotope analysis and the maximum or minimum of the expected range is expressed as Δ^143^Nd or Δ^87^Sr.

The Sr range in the enamel of the Dutch inhabitants (^87^Sr/^86^Sr = 0.70894–0.70982, Fig. 5) is indistinguishable from the previously defined modern Dutch Sr range (^87^Sr/^86^Sr = 0.708–0.710 [2]). The ^143^Nd/^144^Nd ratios from the Dutch individuals from Limburg and Friesland were either compatible (*n* = 13) or lower than (n = 2) the currently defined Dutch geological range (^143^Nd/^144^Nd = 0.51198–0.51217 [57,58]) (Table 1, Fig. 5). The difference of these two individuals compared to the minimum value of the Dutch geological range (^143^Nd/^144^Nd = 0.51198) is Δ^143^Nd = 0.0001–0.0002. The lower Nd isotope ratios recorded for three individuals from Limburg, Friesland and South Holland could potentially be consistent with regional geology, as results as low as ^143^Nd/^144^Nd = 0.5118 are found in the glacial sediment cover derived from geological old terrains in Scandinavia [54,56]. The ^143^Nd/^144^Nd ratios of the individuals from Friesland and Limburg were expected to differ based on the local geology (fluvial and glacial sediments in Friesland and fluvial and loess layers in Limburg), yet overlap in their ^143^Nd/^144^Nd ratio range and median results (Friesland = 0.51182–0.51205, median = 0.51196 and Limburg = 0.51182–0.51208, median = 0.51195). The ^143^Nd/^144^Nd ratios of Friesland and Limburg individuals are lower and less variable than ^143^Nd/^144^Nd ratios of the individuals from North Holland (0.51204–0.51259, median = 0.512189), where local geology consists of fluvial sediments.

Similarly, a local Nd-Sr isotope range was established for the enamel samples from the Caribbean, Columbia and Iceland, using previously published data (see Section 4). The isotope results from the individuals from Grenada, Curaçao, Bonaire, Colombia and Iceland are either (1) consistent with the local geology in both Sr and Nd isotopes (Colombia), (2) consistent with local geology in Nd isotopes but not Sr isotopes, where ^87^Sr/^86^Sr ratios are too high (Grenada, Curaçao and Bonaire) or are (3) incompatible with the local geology in both Sr and Nd isotope ratios (Iceland) (Fig. 6). The elevated ^87^Sr/^86^Sr ratios seen in the individuals from Grenada (0.7078), Curaçao (0.7094) and Bonaire (0.7093) are consistent with the expected contribution from sea-spray (^87^Sr/^86^Sr = 0.7092 [32]) and North African dust (^87^Sr/^86^Sr = 0.715–0.718 [26,61]). The geologically compatible ^143^Nd/^144^Nd ratios of the Caribbean individuals suggest that Nd is less susceptible to sea-spray and dust influences than Sr. Nevertheless, a dust input cannot be ruled out as the North African dust has ^143^Nd/^144^Nd ratios (0.5116–0.5126 [61,62,64]) compatible with the limestone regions of Curaçao and Bonaire (~0.5120 [65]). While the ^143^Nd/^144^Nd ratios of the samples from Curaçao, Bonaire and Colombia reflect the local geology they are indistinguishable from the Dutch population in their Nd isotope ratios as the predicted ^143^Nd/^144^Nd ratios of these countries are similar. The predominantly volcanic origin of Grenada, however, means that the island has a ^143^Nd/^144^Nd ratio distinct from the Dutch geology, and hence the Grenadian individual can be distinguished from the Dutch individuals based on Nd isotope composition. In summary, the ^143^Nd/^144^Nd ratios from the Dutch inhabitants from the provinces of Limburg, Friesland and South Holland as well as the individuals from the Caribbean and Columbia (*n* = 25 out of *n* = 30, 83.3%) are consistent with the hypothesis that the Nd isotope composition of enamel primarily reflects local geology.

Incompatibilities between the Nd isotope ratios of local geology and enamel data were previously reported for 4 individuals from the Netherlands (^143^Nd/^144^Nd ≥ 0.5122, Δ^143^Nd = 0.0002–0.0004 [19]) and are seen in the current study for the Icelandic sample. The Icelandic individual has lower ^143^Nd/^144^Nd and higher ^87^Sr/^86^Sr ratios (0.51189 and 0.70874 respectively) than expected based on the recent volcanic origin of the island (^143^Nd/^144^Nd = ~0.5130 and ^87^Sr/^86^Sr = ~0.70315 [67,68]; i.e., Δ^143^Nd = −0.0011 and Δ^87^Sr = 0.0056). The elevated Sr isotope ratio is consistent with sea-spray contribution, as indicated by other biological datasets from Iceland [69,70]. The Icelandic individual is distinguishable from the ^87^Sr/^86^Sr ratios seen in the enamel of the Dutch inhabitants in this study (^87^Sr/^86^Sr = 0.70894–0.70982). The relatively low Nd isotope ratio cannot be explained solely by local environment factors as Iceland does not receive a major dust input and marine influences should not predominate over the Nd derived from the local geology [51,52]. Incongruent release of Nd during weathering [77] cannot explain the isotopic difference with the volcanic basement as the Icelandic basalt rocks are too young (<5 Ma) to have developed minerals with significant isotopic differences. Although isotopic variations in geologically old terrains (such as Colombia) can be caused by weathering conditions [77,78] the ^143^Nd/^144^Nd ratio of the Colombian individual in this study is consistent with the expected geological isotopic range. The low ^143^Nd/^144^Nd value of the Icelandic individual is isotopically indistinguishable from the lowest ^143^Nd/^144^Nd ratios of modern Dutch enamel (~0.5118), despite major geological differences. Possible explanations for the unexpected Nd isotope values include (1) contact with an anthropogenic Nd source (Section 7.1); or (2) individual dietary preferences for (non-local) food types with different Nd isotope ratios (Section 7.3).

### 7.3 The effect of dietary preferences on ^143^Nd/^144^Nd

The highest ^143^Nd/^144^Nd ratio previously recorded (0.51259 [19]) was a self-reported vegetarian individual from North Holland (11A-15). Two self-reported vegetarian individuals from Friesland, however, do not show elevated ^143^Nd/^144^Nd ratios (39-F8 = 0.51182, 40-F11 = 0.51205). Neodymium isotopes are not expected to be an indicator of trophic level, such as the isotope systems used for diet reconstruction (δ^15^N, ^2^H and ^66^Zn/^64^Zn [[79], [80], [81], [82]]), as the heavy mass of Nd inhibits biological fractionation during the uptake in the human body [47]. Based on the results of this study, it appears that vegetarianism does not result in a coherent isotopic fractionation.

The inconsistencies recorded in ^143^Nd/^144^Nd ratios between human enamel and local geology (*n* = 5 out of n = 30, 16.7%), and unexpected similarities in ^143^Nd/^144^Nd ratios from individuals with variable Dutch geological backgrounds, may be explained by a significant input of non-local food.The globalisation of the food market is an important factor in the isotopic composition of modern human tissues [29]. Both Grenada and Iceland import a large proportion of their food [83,84], which may have lowered the Nd values of the Icelandic individual as primary import countries include the U.S.A., the United Kingdom, Germany, Scandinavian countries, and the Netherlands [83,84]. An alternative explanation is that the low Nd isotope ratio of the Icelandic individual was influenced by the consumption of deep water fish, which are expected to have lower ^143^Nd/^144^Nd values as the Nd isotope composition of North Atlantic seawater ranges between 0.51187 and 0.51213 [52,85]. Unfortunately, the dietary background information of the Icelandic individual was unavailable, so this explanation remains speculative. The elevated ^143^Nd/^144^Nd ratios (≥0.5122) seen in four Dutch individuals may be explained by a reliance on food grown in geological areas with higher ^143^Nd/^144^Nd values (for example the volcanic areas such as East Africa, South America and the Caribbean [60,86,87]). However, the four Dutch individuals with elevated ^143^Nd/^144^Nd ratios had ^87^Sr/^86^Sr ratios compatible with the Dutch Sr range, making a Nd provenance assignment improbable and suggesting that Sr and Nd in enamel may be affected by different environmental factors.

### 7.4 Suggestions for future research

The weak correlation between Nd concentration and Nd isotope composition in Dutch human enamel suggests that at least two Nd components are incorporated into the body, which may include the local geology, anthropogenic Nd, effects of dust and food import. Currently, however, a quantitative interpretation is hampered by the lack of studies on bioavailable neodymium and its uptake into the human body. Further research should include a more comprehensive study of the potential dietary control on Nd isotope ratios by, for example, coupled C and Zn isotope analyses on the enamel of the same dental element or C and N isotope analyses on the collagen of the dentine. This work should be linked to detailed questionnaires to address food consumption, including product origin. To establish a more robust local ^143^Nd/^144^Nd range, subsistence crops and potable water from multiple locations should be analysed for Nd isotope composition and mapped in sufficient numbers (*n* ≥ 20). Furthermore, local Nd pollution sources, such as rare earth element emission, the influence of phosphate-based fertilisers in food production, and the effect of exposure to electronic equipment should be examined. This approach should provide more information on if/how the globalisation of the food market and pollution affect the Nd isotope ratios in modern human enamel. The globalisation effect, or contamination by industrial Nd pollution and fertilisers, is expected to be less influential in archaeological populations that conducted limited long-distance trade and did not use rare earth elements such as Nd on an industrial scale. Archaeological samples, however, have the potential to be affected by diagenesis due to burial. This is particularly the case of buried bone, where the geological Nd signature will overwrite the individual's isotope signature [37,88]. Currently, the contribution of diagenetic Nd and the rate of change is difficult to assess, hampering the isotopic analysis of bone tissue from cold cases in which remains have been buried for several years. Although enamel is a diagenetically resistant tissue, there are known instances where it has been affected by diagenesis [43,89,90]. Work is therefore required to establish the effect of diagenesis on Nd in enamel, with the proviso that if removal of large amounts of the outer enamel layer is needed to avoid diagenetic contamination of archaeological samples [14,91] there may be insufficient sample material available for neodymium analysis.

The analysis of Nd isotopes in human tissues is currently restricted by the large sample sizes required (~200–~1500 mg, >90% of the enamel of a molar) and the need for the latest analytical techniques for TIMS (10^13^ Ω resistors). Hence the use of Nd as part of a multi-isotope provenancing approach will probably be limited to exceptional research questions. On-going developments in mass spectrometry may make it easier to explore the potential of neodymium as a routine tool in human provenance studies. Notably the latest generation multi-collector inducted coupled plasma mass spectrometers (MC-ICP-MS) are producing high quality Hf isotope data (±1 εHf, 2SE) on ~0.3 ng of Hf ([92]). Addition of 10^13^ Ω resistors to such a system has the potential to increase sensitivity further and produce high quality Nd isotope data on <300 pg of Nd.

## 8 Conclusion

This study examined whether Nd isotope composition in human enamel reflects the geological area in which it was formed. For 83.3% of the individuals the variation in Nd isotope composition of human enamel is indistinguishable from the geology of the location where the individual resided when the enamel was formed. This suggests that local geology is the major source of Nd in human dental enamel and in principle that Nd isotopes provide additional information on the mobility profile of an individual, potentially addressing some of the limitations associated with the current isotopic provenancing methods (Sr, Pb, O, H). There are, however, inconsistencies between enamel values and geological ranges in the current dataset. Therefore, further studies are required before Nd provenance studies can be applied to modern enamel samples. Influences on the Nd isotope ratio of human enamel other than local geology, such as dust or anthropogenic sources (electronics, use of fertilisers), and the effect of globalisation of the food market cannot be excluded based on the present dataset. The current uncertainty is due to the relatively limited number of individuals studied from various geological backgrounds and a lack of extensive background data on dietary resources which is required for thorough interpretation. On-going technical developments hold out the prospect that small sample sizes can be measured in the future making the technique more applicable for forensic and archaeological applications.

## Conflicts of interest

There are no conflicts to declare.
